# Supplementary figures and images for: DJ-1 deficiency and aging: dual drivers of retinal mitochondrial dysfunction
Source: bioRxiv. 2025 May 21:2025.05.19.654941. Preprint. [Version 1] doi: 10.1101/2025.05.19.654941 (PMC12139910; doi:10.1101/2025.05.19.654941)

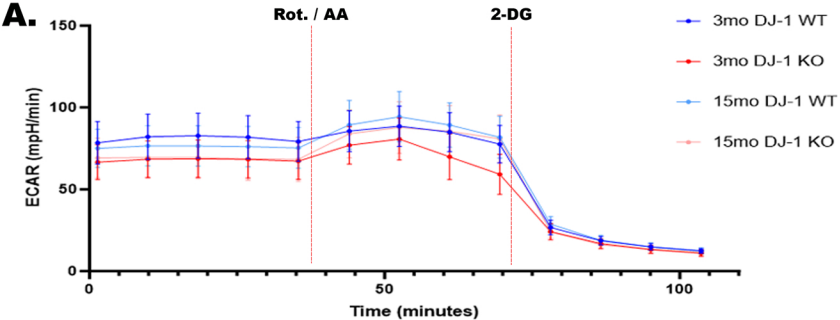

**B.**

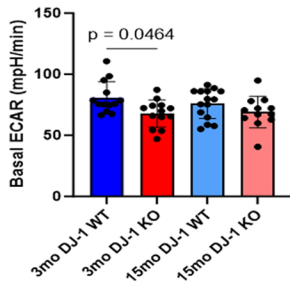

**C.**

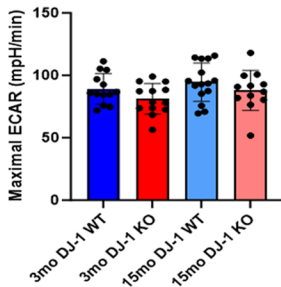

**D.**

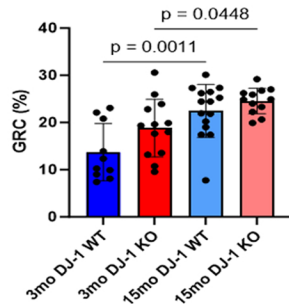

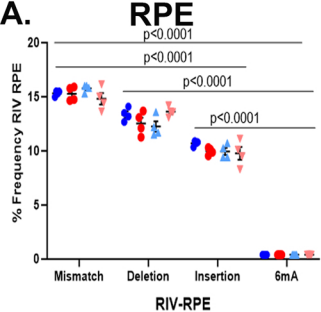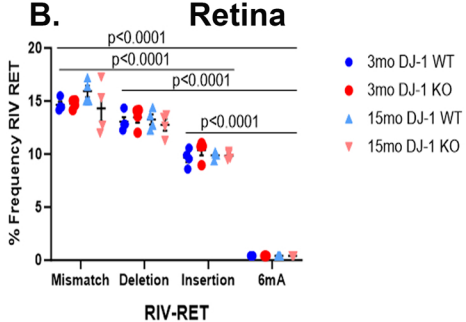

Supplement: Supplement 1 — Figure S1. Effects of aging and DJ-1 loss in ECAR measured by Seahorse analysis. Five measurements were taken before treatment (basal glycolysis), four measurements after 1 μM rotenone/antimycin A injection (maximal glycolysis), and four measurements after 50 mM 2-DG injection (glycolytic reserve capacity). A) Unnormalized ECAR measurements in retinal punches from 3-month-old DJ-1 WT (dark blue), 3-month-old DJ-1 KO (dark red), 15-month-old DJ-1 WT (light blue), and 15-month-old DJ-1 KO (light red) mice. B) Basal ECAR, C) Maximal ECAR, and D) Glycolytic reserve capacity (GRC) of mice described above. Error bar indicates SEM. Data points = technical replicates / individual retina punches from n = 3–5 mice. Statistics: one-way ANOVA; significance: p≤0.05 depicted in the figure. Figure S2. Effects of aging and DJ-1 loss in mtDNA sequencing corresponding to cytb and D-loop (RIV) using nanopore sequencing. Quantification of % frequency for mismatch, deletion, insertion, and 6mA methylation in the A) RPE and B) from 3-month-old DJ-1 WT (dark blue circles), 3-month-old DJ-1 KO (dark red circles), 15-month-old DJ-1 WT (light blue triangles), and 15-month-old DJ-1 KO (light red triangles) mice. Data are represented as mean ± SEM; n=3–4 mice per group. Statistics: two-way ANOVA with Tukey’s multiple comparisons; significance: p≤0.05 depicted in the figure. [file media-1.pdf]
